# The Hidden Metabolites in Glutinous Rice Huangjiu and Their Antioxidant Potential

**DOI:** 10.3390/foods15081386

**Published:** 2026-04-16

**Authors:** Qingxia Zhao, Jingyi Song, Xukai Li, Zhongwei Zhang, Junsong Xiao, Hua Wu, Mingquan Huang

**Affiliations:** 1 Department of Food Science and Engineering, School of Food and Health, Beijing Technology and Business University, Higher Education Garden, Liangxiang, Fangshan District, Beijing 102488, China; 2431031056@st.btbu.edu.cn (Q.Z.); 2050301006@st.btbu.edu.cn (J.S.); 2 Miscellaneous Grain Molecular Breeding Team, College of Agriculture, Shanxi Agricultural University, Taigu 030801, China; xukai_li@sxau.edu.cn; 3 Department of Light Chemical Engineering, School of Light Industry Science and Engineering, Beijing Technology and Business University, Higher Education Garden, Liangxiang, Fangshan District, Beijing 102488, China; zhangzhongwei113@gmail.com; 4 School of Food and Health, Beijing Technology and Business University, Higher Education Garden, Liangxiang, Fangshan District, Beijing 102488, China; xiaojs@th.btbu.edu.cn; 5 School of Light Industry Science and Engineering, Beijing Technology and Business University, Higher Education Garden, Liangxiang, Fangshan District, Beijing 102488, China

**Keywords:** glutinous rice huangjiu, alcoholic liver injury, antioxidant, non-targeted metabolomics

## Abstract

Glutinous rice huangjiu, a non-distilled wine variety unique to China, is rich in nutrients. However, systematic research on the differences in its non-volatile functional components remains scarce, despite these variations being key factors influencing its antioxidant effects. This study employed non-targeted metabolomics to systematically analyze the non-volatile metabolite profiles of 16 glutinous rice huangjiu brands, identifying 1450 metabolites. An alcohol-induced hepatocyte injury model was established, combining cell viability and reactive oxygen species (ROS) level assays to screen for samples (G10 and G11) exhibiting significant efficacy across varying alcohol concentrations. Differential metabolite analysis further identified key bioactive compounds including L-proline, dihydroferulic acid, chalcones, and multiple phenolic derivatives. Using molecular docking technology, we preliminarily revealed that these components may exert antioxidant and hepatoprotective effects either by directly scavenging free radicals or indirectly through mechanisms such as participating in glutathione metabolism and regulating the KEAP1-Nrf2 signaling pathway. This study elucidates the differences among glutinous rice huangjiu at the metabolomic and cellular model levels, providing a scientific basis for evaluating the health benefits and developing new products of huangjiu.

## 1. Introduction

Huangjiu is one of China’s four most famous alcoholic drinks, with a brewing history of over 5000 years [1]. Like wine and beer, huangjiu belongs to the category of fermented alcoholic beverages, featuring a richer composition after fermentation. Glutinous rice serves as the primary ingredient in huangjiu production. Through synergistic fermentation involving Aspergillus oryzae, yeast, and other microorganisms, the process not only preserves essential nutrients like proteins, vitamins, polysaccharides, and minerals [2], but also generates polyphenols and flavonoids, endowing huangjiu with multiple health benefits [3]. The variety of microorganisms, production methods, and raw materials used all work together to give huangjiu its distinct flavors [4]. In China, the medicinal use of huangjiu has a long-standing tradition. Historically known as “clear wine” or “rice wine,” huangjiu has long been used as a medicinal adjunct in China. Its application in traditional Chinese medicine dates back centuries and is documented in the Ming Dynasty’s Compendium of Materia Medica, which notes that “rice wine is specifically used for medicinal purposes”, highlighting its integral role in the preparation of traditional remedies [5]. This stems from huangjiu’s moderate alcohol content and warming nature, which aids in the dissolution of medicinal properties [6]. Moderate consumption of huangjiu is associated with health maintenance [7].

Excessive alcohol consumption can induce oxidative stress, inflammatory responses, and disruptions in autophagy pathways through multiple mechanisms, leading to reversible or irreversible pathological damage to the liver, cardiovascular, nervous, and immune systems [8]. Among these, alcoholic fatty liver disease (ALD) stands as one of the most typical hazards [9]. Years of clinical and experimental research indicate that the core pathogenesis of ALD is closely associated with impaired or insufficient alcohol metabolism, leading to excessive reactive oxygen species (ROS) production [10]. Excessive ROS accumulation triggers oxidative stress [11], subsequently causing lipid peroxidation, DNA damage, disruption of cell membrane integrity, and inflammatory responses. As a key driver, oxidative stress permeates the entire progression of ALD from hepatic steatosis to fibrosis [12]. Consequently, moderate drinking or selecting alcoholic beverages containing active compounds that mitigate alcohol-induced damage has emerged as a trend in healthy drinking practices [13]. Glutinous rice huangjiu, which undergoes no distillation and retains its active components, is rich in antioxidant polyphenols, flavonoids, various amino acids, and bioactive peptides, garnering significant consumer interest and attention [14]. Research indicates [15] that the bioactive compounds enriched during the fermentation of glutinous rice huangjiu have notable health benefits. These components not only effectively counteract oxidative stress damage but also regulate the metabolic balance of key phospholipids in the liver—phosphatidylcholine (PC) and phosphatidylethanolamine (PE) [16]. These mechanisms contribute to its potential in anti-inflammatory and antioxidant effects, as well as in maintaining gut microbiome homeostasis [1].

Although multiple studies have preliminarily revealed correlations between specific components in huangjiu and its health effects, systematic comparative and associative research on the efficacy of huangjiu from different regions and brands in alleviating alcoholic liver damage remains relatively scarce. Therefore, elucidating the components in various huangjiu and their relationship with alcohol-induced damage is an indispensable part of developing health-oriented huangjiu [17]. Non-targeted metabolomics technology based on high-resolution mass spectrometry is currently the standard method for systematically analyzing metabolite compositions in complex food matrices and is also beneficial for studying non-volatile components in huangjiu [18]. However, few studies have reported the integrated identification of components in glutinous rice huangjiu from different geographical regions in China using this technology. In particular, systematic analyses of antioxidant activity and compositional differences among huangjiu from various origins and brands remain scarce.

Therefore, this study employed non-targeted metabolomics to identify non-volatile components in 16 commercial glutinous rice Huangjiu samples sourced from across China. An in vitro model of alcohol-induced hepatocyte injury was established. By comparing the effects of different Huangjiu brands at the same concentration on reactive oxygen species (ROS) scavenging capacity and cell viability in injured hepatocytes, and coupling these findings with differential metabolite analysis, we preliminarily identified bioactive compounds in huangjiu with the potential to mitigate alcoholic liver injury. This research lays the groundwork for further investigation into the nutritional value and health benefits of glutinous rice Huangjiu, offers a scientific approach for establishing health evaluations based on its active constituents, and contributes to the healthy and sustainable development of the Huangjiu industry.

## 2. Materials and Methods

### 2.1. Materials and Reagents

Glutinous rice huangjiu was purchased from 16 different brands (see Supplementary Materials for glutinous rice huangjiu information in Table S1). Due to commercial confidentiality, specific rice varieties for each brand are not disclosed; however, the geographical origins (Shaanxi, Hubei, Jiangsu, Zhejiang, Fujian, and Guangdong) are provided in Table S1. None of the samples were explicitly labeled as autochthonous varieties). Product quality was strictly controlled according to enterprise standards and Chinese national safety standards. HepaRG cells were purchased from Shanghai Fuheng Biotechnology Co., Ltd. (Shanghai, China, Database Name: Cellosaurus, Accession ID: CVCL_9720). The reactive oxygen species (ROS) assay kit was obtained from Beyotime Biotechnology (Shanghai, China). Methanol (chromatographic grade, purity ≥ 99.0%, Thermo Fisher Scientific, Waltham, MA, USA) and 2-Chloro-L-phenylalanine (internal standard reference material, purity 98%, Aladdin Biochemical Technology Co., Ltd., Shanghai, China) were used in this study.

### 2.2. Instruments and Equipment

Freezing centrifuge (Model H1850-R, Hunan Xiangyi Laboratory Instrument Development Co., Ltd., Changsha, China); Vortex mixer (Model BE-2600, Haimen Kylin-Bell Lab Instruments Co., Ltd., Haimen, China); Vacuum Concentrator (Model 5305, Eppendorf, Hamburg, Germany); PTFE Needle Filter (0.22 µm, Jinteng Laboratory Equipment Co., Ltd., Tianjin, China) were used for this study. Both the Vanquish Ultra High Performance Liquid Chromatography System and the Orbitrap Exploris 120 Mass Spectrometer Detector were purchased from Thermo Fisher Scientific (USA).

### 2.3. Sample Preparation

Frozen glutinous rice huangjiu samples were thawed at 4 °C, vortexed for 1 min to mix thoroughly. Then, 100 μL of sample was pipetted precisely into a 2 mL centrifuge tube. Subsequently, 400 μL of pre-chilled methanol solution was added, and the mixture was vortexed for 1 min, then centrifuged at 12,000 rpm at 4 °C for 10 min. The supernatant was collected, evaporated to dryness under nitrogen, and redissolved in 150 μL of 80% methanol aqueous solution (containing 4 ppm 2-chloro-L-phenylalanine). The sample was filtered through a 0.22 μm organic-compatible membrane and then subjected to mass spectrometry analysis. Data acquisition was performed using continuous scanning [19].

### 2.4. Liquid Chromatography and Mass Spectrum Conditions

The LC analysis was performed on a Vanquish UHPLC System (Thermo Fisher Scientific, USA). Chromatographic separation was achieved using an ACQUITY UPLC ^®^ HSS T3 column (2.1 × 100 mm, 1.8 µm) (Waters, Milford, MA, USA). The column temperature was maintained at 40 °C, with a flow rate of 0.3 mL/min and an injection volume of 2 μL. For LC-ESI(+)-MS analysis, the mobile phases consisted of solvent A2 (0.1% formic acid in water, *v*/*v*) and solvent B2 (0.1% formic acid in acetonitrile, *v*/*v*). The elution gradient was set as follows: 0–1 min, 8% B2; 1–8 min, 8–98% B2; 8–10 min, 98% B2; 10–10.1 min, 98–8% B2; 10.1–12 min, 8% B2. For LC-ESI(−)-MS analysis, the mobile phases consisted of solvent A3 (5 mM ammonium formate in water) and solvent B3 (acetonitrile). The same elution gradient as the positive mode was applied: 0–1 min, 8% B3; 1–8 min, 8–98% B3; 8–10 min, 98% B3; 10–10.1 min, 98–8% B3; 10.1–12 min, 8% B3 [20].

Mass spectrometric detection was performed on an Orbitrap Exploris 120 mass spectrometer (Thermo Fisher Scientific, USA) equipped with an ESI ion source. Data were acquired in the full MS/data-dependent MS/MS (Full MS-ddMS^2^) mode, enabling simultaneous collection of MS1 and MS/MS spectra. The source and acquisition parameters were set as follows: sheath gas pressure, 40 arbitrary units (arb); auxiliary gas flow, 10 arb; spray voltage, 3.50 kV for ESI(+) and −2.50 kV for ESI(−); capillary temperature, 325 °C. For MS1 acquisition: mass range, *m*/*z* 100–1000; resolving power, 60,000 FWHM. For MS/MS acquisition: 4 data-dependent scans per cycle; resolving power, 15,000 FWHM; normalized collision energy (NCE), 30%; dynamic exclusion time, automatic [21].

### 2.5. Cell Culture and Cell Viability Assay

The HepaRG cells were cultured in complete DMEM medium (containing 10% fetal bovine serum and 5% dual antibiotics) under standard conditions at 37 °C in a 5% CO_2_ incubator. Upon reaching the plateau phase (approximately 80–90% confluence), the old medium was removed, cells were washed twice with PBS and then digested with 0.25% EDTA-trypsin for passage or counted for subsequent experiments.

HepaRG cells (5 × 10^3^ cells/well) were seeded into 96-well plates and cultured for 24 h at 37 °C in a 5% CO_2_ incubator. Subsequently, cells were treated with 200 mM or 400 mM ethanol, or 16 different types of glutinous rice wine at corresponding ethanol concentrations for 12 h. A volume of 100 μL of cell culture medium was replaced with 10 μL of CCK-8 solution in each well, incubated for 0.5–1 h, then the absorbance at 450 nm was measured using a microplate reader (Männedorf, Switzerland).

### 2.6. ROS Level Detection

HepaRG cells (2 × 10^4^ cells/well) were seeded into black 96-well plates and cultured at 37 °C in a 5% CO_2_ incubator for 24 h. Cells were treated with 200 mM or 400 mM ethanol, or 16 different brands of glutinous rice wine corresponding to the ethanol levels for 12 h. Subsequently, ROS levels in the samples were measured according to the instructions from the Beyotime Reactive Oxygen Species (ROS) Detection Kit (Shanghai, China).

### 2.7. Molecular Docking

Molecular docking analysis was performed using AutoDock Vina 1.2.0. The 3D structures of characteristic components were obtained from the PubChem database (minimized and saved in PDB format). The crystal structure of protein 4CXT, representing the KEAP1-BTB domain complexed with CDDO, was downloaded from the Protein Data Bank. Water molecules and ligands were removed using PyMOL 2.5, followed by hydrogenation. Docking parameters were set as follows: the grid box center covered the key binding site of Keap1 (near Cys151 residue, grid size 20 × 20 × 20 Å, spacing 0.375 Å), with exhaustiveness: 20. Docking results were analyzed using discovery studio 2019 to examine hydrogen bonds and hydrophobic interactions. Binding energies ≤ −5.0 kcal/mol were considered significant interactions. All docking experiments were independently repeated three times [22].

### 2.8. Data Processing and Statistical Analysis

#### 2.8.1. Data Preprocessing

Raw mass spectrometry data files were converted to mzXML format using the MSConvert tool in the ProteoWizard software package (v3.0.8789). Peak detection, filtering, and alignment were performed using the XCMS package in R, with the following parameters: bw = 2, ppm = 15, peakwidth = c(5, 30), mzwid = 0.015, mzdiff = 0.01, method = centWave, which generated a list of quantified metabolite features. Support vector regression (SVR) correction based on quality control (QC) samples was applied to eliminate systematic errors. Finally, only metabolite features with a coefficient of variation (CV) < 30% in QC samples were retained for subsequent statistical analysis.

#### 2.8.2. Metabolite Identification

Metabolite identification was performed using Progenesis QI v3.0 software (Waters Corporation, Milford, CT, USA), searching against public databases (HMDB: http://www.hmdb.ca/ (accessed on 25 October 2025); METLIN: https://metlin.scripps.edu/ (accessed on 25 October 2025)) and an in-house database. Molecular formulas were predicted based on MS1 precursor ion *m*/*z*, adduct ions, and isotope peaks, with a 10 ppm mass tolerance for database matching. MS/MS spectra of each precursor ion were matched with theoretical spectra via a modified weighted cosine similarity scoring algorithm: putative metabolites with scores ≥ 30 were candidate matches, and those with experimental scores ≥ 35 and theoretical scores ≥ 40 were high-confidence annotations. Metabolites were annotated using KEGG and HMDB databases, then manually inspected to remove suspected exogenous or unreliable compounds. Key differential metabolites were further confirmed by comparing their MS/MS fragment patterns with reference spectra to ensure identification confidence.

#### 2.8.3. Statistical Analysis

Principal Component Analysis (PCA) was performed using SIMCA multivariate data analysis software (v18.0.0.372, developed by Umetrics, Umeå, Sweden). Hierarchical cluster analysis results for samples and metabolites were visualized as heatmaps with dendrograms. For the two-group analysis, differential metabolites were screened based on Projected Variable Importance (VIP) scores (VIP ≥ 1) and Fold Change (FC) (FC > 2). VIP values were extracted from orthogonal partial least squares discriminant analysis (OPLS-DA) results (including score plots), and data underwent log2 transformation for volcano plot visualization. Using the MicroBioinformatics Cloud Platform (https://www.bioinformatics.com.cn), standardized matrix data were downloaded for Mfuzz clustering analysis. All figures were generated using Origin 2021 to ensure data visualization adheres to academic standards.

## 3. Results

### 3.1. Non-Targeted Metabolomics Identification of Non-Volatile Metabolites in Glutinous Rice Wine

**Figure 1:**
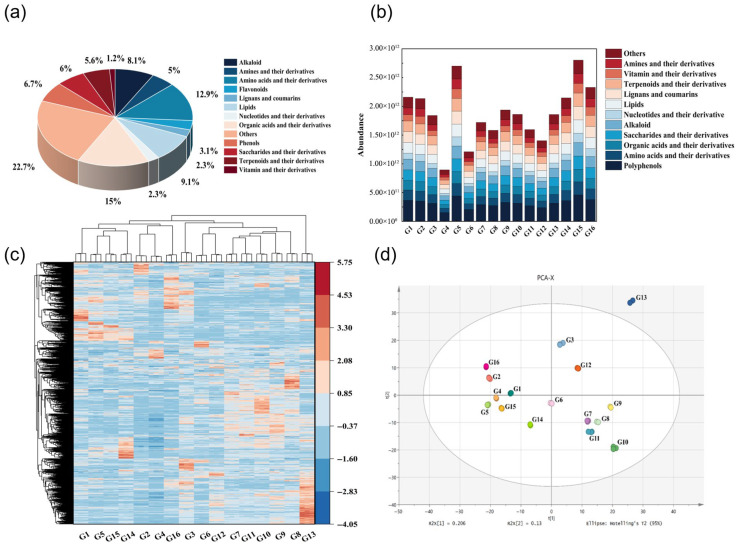
Metabolomic Analysis of Glutinous Rice huangjiu Samples. From left to right and top to bottom: (**a**) Proportional distribution of metabolites in glutinous rice huangjiu, (**b**) Peak area histogram of metabolites in glutinous rice huangjiu, (**c**) Clustering heatmap of metabolites in glutinous rice huangjiu, (**d**) Distribution in the two-dimensional score plot of principal component analysis for glutinous rice huangjiu. Overlapping circles of the same color in the figure indicate three sets of duplicates.

Non-targeted metabolomics analysis was conducted on 16 different brands of glutinous rice huangjiu. Results revealed a total of 1450 non-volatile metabolites. The number and proportion of metabolites across all categories are shown in Figure 1a. All metabolites were classified into 12 categories. The most abundant categories across all glutinous rice huangjiu samples were organic acids and their derivatives (217 types), amino acids and their derivatives (187 types), polyphenols (142), lipids (133), and alkaloids (118). Among polyphenolic metabolites, phenols (97) and flavonoids (45) were identified.

By conducting a relative quantitative comparison of various metabolite types across 16 different brands of glutinous rice huangjiu, as shown in Figure 1b, it was found that all 12 metabolite categories were detected in the 16 sample groups. Among these, amino acids and their derivatives, alkaloids, organic acids and their derivatives, lipids, and phenolic compounds were particularly prominent in quantity compared to other categories. Sample G4 exhibited the lowest metabolite content across all categories, while samples G5 and G15 showed higher metabolite content overall. Among all samples, the peak area summation of polyphenolic metabolites consistently exceeded that of other metabolite categories, with relatively uniform peak area distributions observed for other metabolite groups.

The row and column clustering results for 16 glutinous rice huangjiu samples are shown in Figure 1c. The G13 sample group forms a distinct cluster, showing significant differences from all other groups. G1, G5, G15, G14, G2, G4, and G16 cluster together as one group, while G3, G6, G12, G7, G11, G10, G9, and G8 form another cluster. Alkaloids are relatively abundant and present in higher relative concentrations within these clusters, particularly in G10 and G8. In contrast, amino acids and their derivatives show relatively low abundances, while polyphenols and flavonoids are relatively abundant in the G8, G9, G10, and G11 groups. The relatively abundant polyphenols in these groups include ferulic acid, pinoresinol, and dihydroferulic acid, while the flavonoids include quercetin and the isoflavonoid glycoside (−)-medicarpin-3-O-glucoside.

To explore variations and classification of non-volatile metabolites in glutinous rice huangjiu samples from different brands, principal component analysis (PCA) was performed. As shown in Figure 1d, the PCA biplot similarly revealed clustering of the three biological replicates within each group. Results indicate that the first principal component (PC1) and second principal component (PC2) explain 20.6% and 13% of the variance, respectively. Samples exhibit a distinct separation trend, with the 16 glutinous rice huangjiu samples scattered across the PCA plane, suggesting substantial differences in metabolites between samples. Notably, sample G13 lies outside the PCA plane, indicating significant differences in metabolite profiles compared to other samples. Samples G3 and G12 were distributed in the first quadrant; samples G1, G2, and G16 were distributed in the second quadrant; G4, G5, G6, G14, and G15 samples were distributed in the third quadrant; the remaining samples (G7, G8, G9, G10, and G11) were located in the fourth quadrant and exhibited a more clustered distribution on the PCA plane. This aligns with the results from the metabolite clustering heatmap analysis in Figure 1c, indicating that these glutinous rice huangjiu samples share commonalities in their metabolite profiles with relatively minor differences.

### 3.2. Effects of Semi-/Non-Volatile Components in Glutinous Rice Wine from Different Origins on Alcohol-Induced Hepatocyte Damage

HepaRG cells were treated for 12 h with either 200/400 mM alcohol alone or with the semi-/non-volatile fractions from rice wine containing equivalent alcohol concentrations. Cell viability and reactive oxygen species (ROS) levels were compared across samples under 200/400 mM conditions.

**Figure 2:**
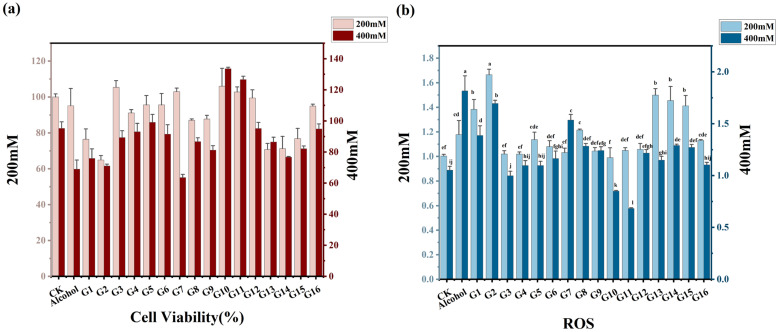
Effects of glutinous rice huangjiu on (**a**) survival rate and (**b**) ROS levels in alcohol-induced HepaRG cells. Lowercase letters (a–j): A *p*-value of less than 0.05 indicates a statistically significant difference between two groups. Groups labeled with the same letter show no significant difference (*p* > 0.05).

As shown in Figure 2a, under the 200 mM treatment condition, the cell survival rate in the alcohol-treated group was 95.19%. However, among samples treated with 200 mM and supplemented with different alcoholic beverage resuspension solutions, survival rates exhibited significant variability. Specifically, cell survival rates exceeded 100% in samples treated with G3, G7, G10, and G11 reconstituted solutions, whereas the G2 reconstituted solution group exhibited a survival rate of only 64.94%, falling below 80%. Under 400 mM treatment conditions, the alcohol-treated group showed a notably lower survival rate of 69.03%. Cell survival rates after treatment with G3, G4, G5, and G6 samples were all higher than those in the alcohol-treated group. Notably, the survival rates in the G10 and G11 sample groups reached 135.51% and 126.55%, respectively, indicating significantly higher survival rates and the most effective treatment outcomes.

As shown in Figure 2b, under the 200 mM treatment condition, the ROS content in the G2 treatment group was the most prominent, reaching 1.41 times that of the alcohol group. Compared to the alcohol-treated group, ROS levels in the G3, G4, and G7 sample treatment groups decreased by approximately 13.42%, 13.45%, and 12.4%, respectively. Additionally, ROS levels in the G10 sample treatment group decreased by approximately 15.93% compared to the alcohol-treated group and by approximately 1.3% compared to the normal cell group. Under 400 mM treatment conditions, ROS levels in all 16 treated cell groups were lower than the alcohol-treated group. Cells treated with G1, G2, and G7 samples exhibited ROS levels approximately 1.36, 1.67, and 1.51 times higher than the blank control group, respectively, showing a more pronounced effect. Other treatment groups did not show significant increases compared to the blank control group. Additionally, ROS levels in the G10 and G11 treatment groups decreased by approximately 19% and 35%, respectively, compared to the blank group. These results indicate that G10 and G11 exhibited the greatest reduction in cellular ROS levels. Since G2 exhibited the highest ROS levels and lowest cell viability among all samples, it was used as a control to identify metabolites potentially associated with superior protective effects by comparing the best-performing (G10/G11) and worst-performing (G2) samples.

### 3.3. Screening Results for Metabolites That May Cause Differences in ROS Accumulation

**Figure 3:**
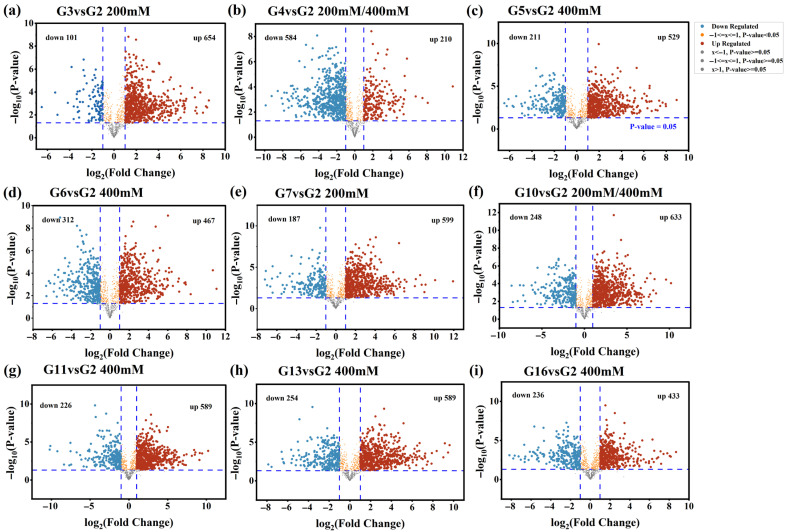
Volcano plots of differentially expressed metabolites in the 200 mM and 400 mM groups (set at *p* < 0.05 and FC > 2). Blue dots and red dots represent downregulated and upregulated metabolites, respectively, while gray dots indicate metabolites without significant differences (In the figure, “G3 vs. G2,” for example, refers to the metabolite volcano plot of the G3 treatment group compared to the G2 group; “200 mM” indicates the group selected at an ethanol concentration of 200 mM; and “200 mM/400 mM” indicates the group selected for both of these concentrations).

To further investigate the correlation between the effects of non-volatile metabolites from different glutinous rice huangjiu on cellular ROS production at varying alcohol concentrations, the G2 group under 200 mM and 400 mM conditions served as the control. Comparisons were performed based on the principle of FC > 2 and *p* < 0.05. As shown in Figure 3a,b,e,f, a total of 654 (G3 vs. G2), 210 (G4 vs. G2), 599 (G7 vs. G2), and 633 (G10 vs. G2) metabolites were identified as upregulated, while 101 (G3 vs. G2), 584 (G4 vs. G2), 187 (G7 vs. G2), and 248 (G10 vs. G2) were downregulated metabolites. As shown in Figure 3b–d,f–i, a total of 654 (G3 vs. G2), 210 (G4 vs. G2), 529 (G5 vs. G2), 633 (G10 vs. G2), 589 (G11 vs. G2), and 433 (G16 vs. G2) were identified as up-regulated metabolites, while 211 (G5 vs. G2), 226 (G11 vs. G2), and 236 (G16 vs. G2) were down-regulated. These primarily comprised amino acids, organic acids, and polyphenolic compounds. Overall, most branded glutinous rice huangjiu samples exhibited upregulation of numerous non-volatile metabolites, whereas G4 samples showed a predominant downregulation trend in most non-volatile metabolites.

**Figure 4:**
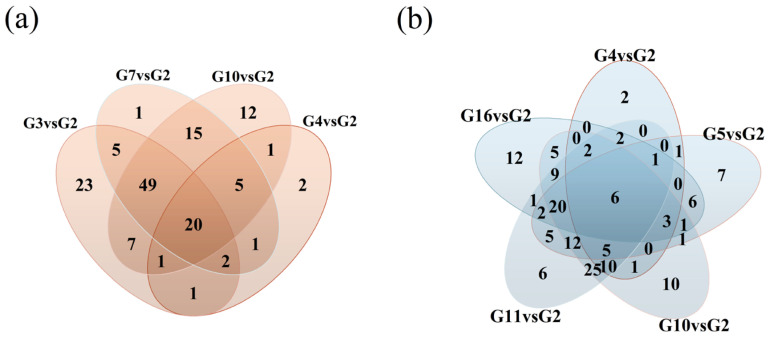
Venn diagram of differentially metabolized compounds at 200 mM (**a**) and 400 mM (**b**) concentrations.

Based on the volcano plot analysis results, differential metabolites were screened using the criteria of VIP ≥ 1, *p* < 0.05, and FC > 2. Comparisons of differential metabolites between G3, G4, G7, G10, and G2 revealed both shared and unique non-volatile differential metabolites across comparison groups. As shown in Figure 4a, G3 samples exhibited 108 differentially expressed metabolites, G4 samples showed 33 differentially expressed metabolites, G7 samples contained 98 differentially expressed metabolites, and G10 samples contained 110 differential metabolites. Among these, 22 differential metabolites were common between G3, G4, and G7 groups, while 20 differential metabolites were common among G3, G4, G7, and G10 groups. To further screen the 20 common differential metabolites, they were ranked by fold change (FC) values. The selected metabolites were then subjected to correlation analysis with cellular ROS data. Substances showing a significant negative correlation with ROS scavenging capacity and high FC values were identified. Through this process, two common differential metabolites with notably high FC values in the G10 group were preliminarily determined: L-Proline and Isoleucyl-Glutamine. Compared to G3, G4, and G7 groups, L-proline and isoleucyl-glutamine exhibited the highest FC values and were up-regulated in the G10 group. This finding strongly correlates with G10 demonstrating optimal ROS scavenging capacity and cell viability under 200 mM conditions.

Comparisons of differentially expressed metabolites between G3, G4, G5, G10, G11, G16, and G2 relative to G2 are shown in Figure 4b. Results indicate that the number of differentially expressed metabolites in G3, G4, G10 share the same number of differential metabolites as in the 200 mM group. The G5 sample exhibits 70 differential metabolites, the G11 sample shows 106 differential metabolites, and the G16 sample contains 70 differential metabolites. G4, G5, and G16 exhibited consistent expression trends, sharing 10 common differentially expressed metabolites. Further screening of these 10 metabolites identified 6 significantly common differentially expressed metabolites. Based on FC value ranking, dihydroferulic acid was preliminarily determined as the common metabolite significantly negatively correlated with ROS scavenging capacity in the G11 group and exhibiting a relatively significant FC value. Compared to G4 and G5, and G16. This finding aligns with the optimal ROS scavenging capacity and cell viability observed in G10 and G11 under 400 mM conditions.

### 3.4. Mfuzz Clustering Results of Non-Volatile Compounds and ROS Accumulation

**Figure 5:**
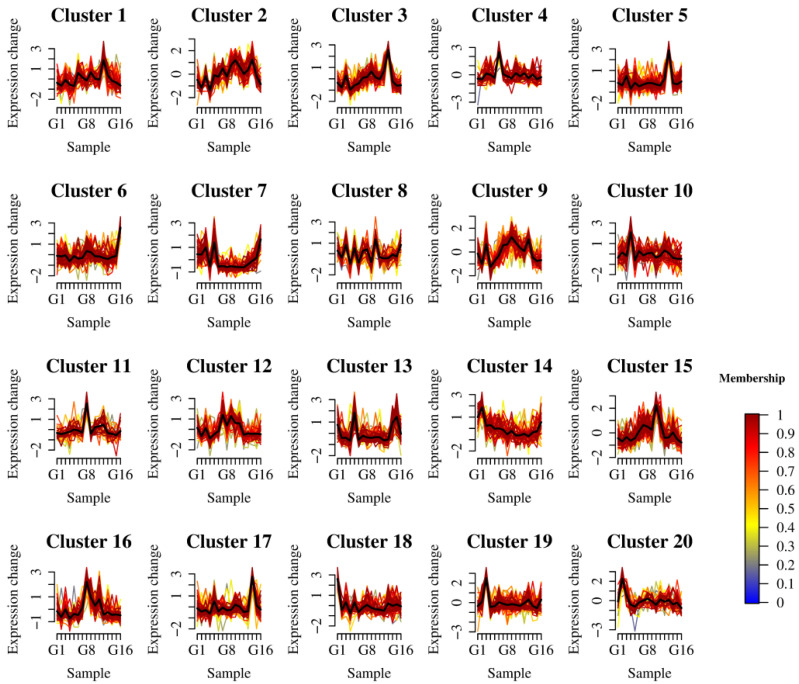
Mfuzz clustering analysis of ROS200 and ROS400 with related substances (ROS200 and ROS400 denote free radical ROS levels under 200 and 400 mM conditions. The black line in the figure represents the centerline).

Based on the aforementioned experimental findings, we categorized metabolites according to their concentration trends across different samples. Using Mfuzz clustering analysis to examine the relationship between 1450 non-volatile metabolites and ROS content yielded the results shown in Figure 5. The results indicate that these 1450 metabolites were grouped into 20 distinct clustering patterns, with substances within the same cluster exhibiting correlations. A total of 40 substances associated with ROS levels under 200 mM treatment were found in Cluster 8, while 47 substances linked to ROS levels under 400 mM treatment were identified in Cluster 14. Further analysis based on clustering results identified metabolites with Membership values > 0.6. Findings indicate 18 core metabolites correlated with ROS levels at 200 mM and 28 core metabolites correlated with ROS levels at 400 mM. These substances primarily include flavonoids, phenolics, and lipids, which research has demonstrated possess potent antioxidant effects.

**Figure 6:**
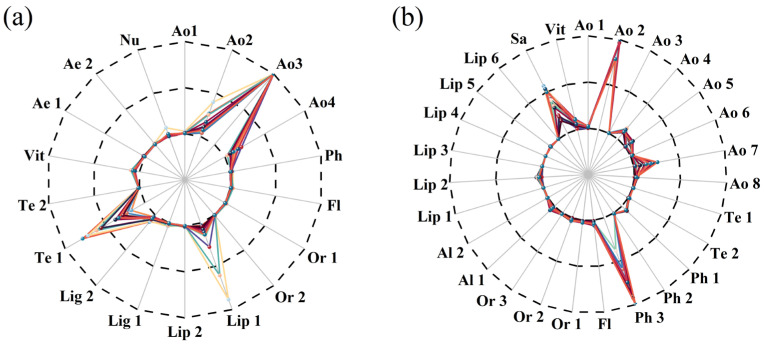
Radar charts of core substances related to ROS200 (**a**) and ROS400 (**b**) (Different line colors indicate different groups. substance names are represented by abbreviations; specific names are listed in Table S2 of the Supplementary Materials).

Differential analysis of core substances related to ROS200 and ROS400 is shown in Figure 6a,b. Under ROS200 conditions, Ao3, Lip1, and Te1 exhibited prominent differences. Under ROS400 conditions, Ao2, Ao4, Ao5, Ao7, Ph3, and Sa showed significant differences among the 28 core substances. Further analysis revealed that Ao2 (Methionine), listed as methionine in the table, is a key precursor for glutathione synthesis. Ao7 (L-γ-glutamyl peptides), a glutathione derivative, may exert potent antioxidant effects via the glutathione pathway. Ph3 ((E)-3-(2,3-Dihydroxyphenyl)-2-propenoic acid) is a cinnamic acid derivative of the catechol class. The catechol structure is a classic potent antioxidant group that efficiently neutralizes free radicals by donating hydrogen atoms, delivering strong antioxidant effects.

### 3.5. Molecular Docking Prediction Results for Antioxidant Mechanisms of Core Differential Metabolites

To further investigate the potential mechanisms underlying the antioxidant activity of these key differentially expressed compounds, we examined the three compounds identified in Section 3.4 as exhibiting the most significant differences (methionine, L-γ-glutamylpeptide, and (E)-3-(2,3-dihydroxyphenyl)-2-propenoic acid), the three compounds associated with differences in reactive oxygen species (ROS) accumulation at a concentration of 400 mM in Section 3.3 (dihydroferulic acid, L-proline, and isoleucyl-glutamine), and the protein 4CXT (PMID: 24896564), which represents the KEAP1-BTB domain-CDDO complex. The results are shown in Table 1 below, with binding free energies (ΔG) ranging from −6.606 to −3.567 kcal/mol. Among these, L-γ-glutamyl peptide exhibited the strongest binding affinity at −6.606 kcal/mol, while Methionine showed the weakest at −3.567 kcal/mol. This result aligns closely with literature reports on the biological function of glutathione (GSH) as a natural ligand for KEAP1, suggesting its potential as an effective KEAP1 ligand. In contrast, Methionine’s weakest binding affinity (−3.567 kcal/mol) indicates difficulty in forming stable complexes with 4CXT at physiological concentrations, suggesting it may exert antioxidant effects through alternative pathways.

**Figure 7:**
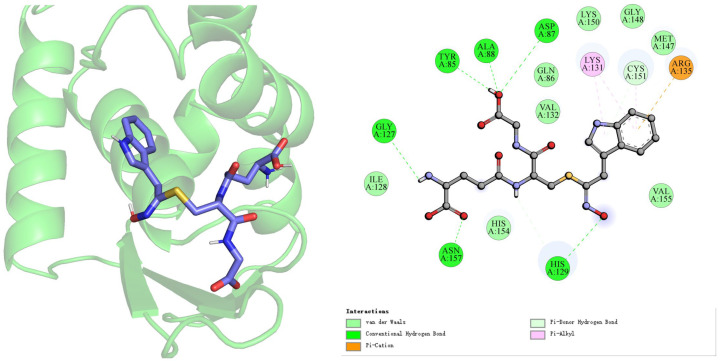
Molecular docking results of L-γ-glutamyl peptide with the KEAP1-BTB domain (PDB: 4CXT).

As shown in Figure 7, molecular docking analysis of L-γ-glutamyl peptide with the KEAP1-BTB domain (PDB: 4CXT) indicates that the compound stably binds within the BTB domain pocket of KEAP1. This pocket also serves as the binding site for the known NRF2 activator CDDO (PMID: 24896564). L-γ-glutamyl peptide uses Tyr85 as its binding core, forming hydrogen bonds with His129, Asp87, Ala88, and others. It binds to Arg135 via strong π-cation interactions while maintaining close π-alkyl contact with Lys131. This binding pattern indicates that the ligand noncovalently occupies both the NRF2-competitive interface and covalent modification site of KEAP1, acting as a potentially potent reversible inhibitor. It occupies the classic pocket of the known activator CDDO, thereby exerting antioxidant effects.

Molecular docking between Methionine and ROS was performed to investigate whether it exerts antioxidant effects through direct interaction with ROS. Results indicate that Methionine can directly interact with ROS through its sulfhydryl group (-S-CH_3_). In the docking model, the sulfur atom of Methionine acts as a nucleophilic center, forming a stable binding conformation with the oxygen atom of H_2_O_2_, with a binding energy of approximately −50.74 kcal/mol. This suggests a strong spontaneous binding tendency between the two. From a reaction mechanism perspective, the sulfhydryl group of methionine can be oxidized by ROS, sequentially generating methionine sulfoxide and methionine sulfone. The simplified process of this reaction can be represented as:

Met-S-CH_3_ + H_2_O_2_ → Met-SO-CH_3_ + H_2_O (1)

This finding suggests that methionine, as a direct ROS scavenger, may exert antioxidant effects by consuming free radicals through covalent binding and redox reactions. This discovery provides molecular-level evidence elucidating the antioxidant mechanism of organic sulfur-containing amino acids in glutinous rice huangjiu. It further supports the hypothesis that bioactive components in fermented foods maintain redox balance through direct scavenging, pathway regulation, or dual synergistic modes.

## 4. Discussion

Based on the traditional understanding of huangjiu as a substance with both medicinal and dietary properties, as well as its characteristic as a complex multicomponent system [23], this study is the first to integrate non-targeted metabolomics with cellular models to systematically elucidate the potential material basis and mechanisms through which different brands of glutinous rice huangjiu alleviate alcoholic hepatocyte injury. Our findings indicate that not all huangjiu products exert the same cellular protective effects, suggesting that their health benefits are product-specific. This specificity is likely attributable to the distinct metabolite profiles generated during fermentation [24]. Cellular models were employed to evaluate the effects of different glutinous rice huangjiu samples on alcohol-induced oxidative damage markers in HepaRG cells. Experimental results revealed marked differences in cell survival rates and reactive oxygen species (ROS) levels among alcohol-treated groups receiving different glutinous rice huangjiu brands. Specifically, the G10 and G11 sample treatment groups exhibited higher cell survival rates and lower ROS accumulation, suggesting that certain components in these corresponding samples may possess regulatory potential against alcohol-induced cellular oxidative stress.

Further comparative analysis via non-targeted metabolomics revealed that the aforementioned cellular phenotypic differences correlate with the compositional characteristics of certain non-volatile metabolites in the samples. Particularly in G10 and G11, components such as L-proline [25], dihydroferulic acid [26], and chalcones [27] were relatively enriched. These components have been widely reported in existing studies to possess antioxidant activity. Research indicates that the functional amino acid L-proline maintains redox and immune homeostasis [28], demonstrating its ability to directly scavenge the large amount of ROS generated during alcohol metabolism, thereby reducing lipid peroxidation and hepatocyte membrane damage [29]. Additionally, ferulic acid and its analogs can significantly scavenge free radicals, downregulate pro-inflammatory markers, and inhibit inflammatory enzymes, thereby attenuating hepatocyte injury caused by cytotoxicity and apoptosis [30,31]. Chalcones exert anti-inflammatory effects through indirect mechanisms by modulating the Keap1/Nrf2-ARE pathway, a key regulator of oxidative stress and inflammation. This leads to reduced reactive oxygen species (ROS) levels and decreased NF-κB activity, while simultaneously activating the expression of multiple antioxidant and anti-inflammatory genes, as well as genes involved in the clearance of damaged proteins [32]. These genes also encode enzymes that produce glutathione (GSH), the primary intracellular antioxidant, thereby regulating cellular responses to oxidative stress and inflammation [33,34]. It is worth noting that the antioxidant activity of polyphenolic compounds in huangjiu (such as dihydroferulic acid and chalcones) depends not only on their ability to directly scavenge free radicals but may also enhance the expression of downstream antioxidant enzymes by activating the Nrf2 pathway, thereby synergistically boosting cellular antioxidant defense capabilities [35]. Similarly, the hepatoprotective effects of non-volatile components in Huangjiu are believed to be closely related to their regulation of hepatic metabolic enzyme activity, alleviation of endoplasmic reticulum stress, and suppression of inflammatory responses, suggesting that their mechanism of action exhibits synergistic characteristics involving multiple targets and pathways [36]. These findings provide theoretical support for the synergistic effects of various bioactive components in this study and suggest that different components may jointly exert antioxidant and hepatoprotective functions through complementary mechanisms.

To further explore the potential mechanisms of action of these bioactive compounds, molecular docking analysis revealed that L-proline exhibits weak binding affinity to the BTB domain of KEAP1, a core regulatory protein involved in antioxidant defense, with a binding energy of −4.005 kcal/mol. The antioxidant activity of L-proline likely depends primarily on its direct chemical scavenging of free radicals and its role in GSH synthesis metabolism [37], rather than direct regulation of the KEAP1-Nrf2 signaling pathway. Isoleucyl-Glutamine [38] exhibits stronger binding affinities, potentially exerting antioxidant effects by directly targeting the KEAP1-Nrf2 pathway. Although Methionine exhibits significant in vitro antioxidant activity, its weak binding to KEAP1 (−3.567 kcal/mol) suggests its antioxidant effects are unlikely to be primarily mediated through the KEAP1-Nrf2 pathway. This finding is consistent with the established antioxidant mechanism of methionine: its sulfhydryl group can be directly oxidized by ROS into a sulfoxide, thereby protecting more critical methionine residues in proteins [39]. Its antioxidant action in vivo manifests primarily as direct chemical scavenging of free radicals rather than signaling pathway regulation. Furthermore, as a complex multicomponent system, the overall antioxidant effect of glutinous rice huangjiu may result from the combined actions of multiple constituents. Whether synergistic or antagonistic effects exist among these components warrants further investigation.

In addition to the aforementioned polyphenols and amino acids, this study identified 118 alkaloids in glutinous rice wine, primarily consisting of betaine and its related derivatives. Experimental results indicate that alkaloids have a relatively high abundance among various metabolites, and existing research suggests that they also possess potential antioxidant and hepatoprotective activities. Betaine is a choline-derived metabolite and methyl donor with antioxidant, anti-inflammatory, and osmoprotective properties [40]. Recent studies have found that low betaine levels are associated with more severe organ damage, while betaine supplementation can effectively prevent the onset of alcohol-related liver disease (ALD) [40]. Its mechanism of action involves regulating the gut microbiota, maintaining intestinal barrier integrity, and alleviating hepatic inflammatory responses by modulating the gut-liver axis. Furthermore, combinations of methyl donors (such as betaine, methionine, and folate) have been shown in animal models to significantly improve metabolic and histological parameters in alcoholic steatohepatitis [41]. It should be noted that alkaloids were not significantly enriched in the differential metabolite screening in this study, which may explain why their differential contribution was less pronounced than that of polyphenols and amino acids. However, given that alkaloids may exert their effects through synergistic interactions with other components, their role in the health benefits of Huangjiu warrants further investigation.

In summary, this study not only correlates the non-volatile components of glutinous rice huangjiu with its antioxidant capacity at the metabolite abundance level but also delves into molecular interactions through computational modeling to predict potential differences in the mechanisms of action among various bioactive compounds. These findings offer new insights into understanding the health benefits of huangjiu and lay a solid theoretical foundation for the targeted development of functional products with clearly defined efficacy markers. Future research may directly validate these computationally predicted mechanisms through approaches such as gene knockout and reporter gene experiments.

## 5. Conclusions

This study employed non-targeted metabolomics to systematically analyze the non-volatile metabolite profiles of glutinous rice huangjiu from 16 brands, identifying a total of 1450 metabolites across multiple categories including amino acids, polyphenols, flavonoids, and organic acids. By establishing an alcohol-induced hepatocyte injury model and combining cell viability with ROS level detection, glutinous rice huangjiu samples (G10 and G11) exhibiting significant protective effects under both 200 mM and 400 mM alcohol induction were selected. Differential metabolite analysis further identified key bioactive compounds closely associated with antioxidant activity, including L-proline, methionine, as well as polyphenolic derivatives such as dihydroferulic acid and chalcones. Molecular docking analysis suggested these compounds may exert antioxidant effects through direct radical scavenging, participation in glutathione metabolism, or regulation of signaling pathways such as KEAP1-Nrf2, though their specific mechanisms differ. This study represents the first integrated metabolomics and cellular model analysis to reveal the efficacy differences and underlying mechanisms of glutinous rice huangjiu from distinct origins in mitigating alcoholic liver injury. It provides systematic scientific evidence and methodological support for elucidating the health effects of traditional fermented alcoholic beverages and developing huangjiu products with clearly defined functional biomarkers.

## Figures and Tables

**Table 1:** Molecular Docking Binding Energy of Six Antioxidants with 4CXT.

NO.	Metabolite Names	Binding Energy (kcal/mol)
*1*	Dihydroferulic Acid	−5.084
*2*	L-Proline	−4.005
*3*	Isoleucyl-Glutamine	−4.685
*4*	Methionine	−3.567
*5*	L-γ-glutamyl peptides	−6.606
*6*	(E)-3-(2,3-Dihydroxyphenyl)-2-propenoic acid	−4.612

## Data Availability

The original contributions presented in this study are included in the article/Supplementary Materials. Further inquiries can be directed to the corresponding authors.

## Supplementary Materials

The following supporting information can be downloaded at: https://www.mdpi.com/article/10.3390/foods15081386/s1, Table S1. Detailed information of the 16 commercial glutinous rice Huangjiu samples, including brand names, geographical origins, rice varieties, and alcohol content. (Specific rice varieties are not disclosed due to commercial confidentiality; geographical origins are provided instead). Table S2. Full names of the abbreviated compounds presented in Figure 6 (Ao2, Ao3, Ao4, Ao5, Ao7, Lip1, Te1, Ph3, Sa, etc.), along with their metabolite categories.

## Abbreviations

The following abbreviations are used in this manuscript:

ROS	Reactive Oxygen Species
ALD	Alcoholic Liver Disease
PC	Phosphatidylcholine
PE	Phosphatidylethanolamine
FC	Fold Change
